# 501 The Effect of Renal Failure on Acute Burn Mortality at a Tertiary Care Burn Center

**DOI:** 10.1093/jbcr/irae036.136

**Published:** 2024-04-17

**Authors:** Ashley Levine, Jamie L Hollowell, Benjamin Bodek, Chris B Agala, Eli Maxwell, Lori Chrisco, Robert W Matthews, booker King, Felicia Williams

**Affiliations:** University of North Carolina, Durham, NC; University of North Carolina, Chapel Hill, NC; University of North Carolina Chapel Hill, carrboro, NC; UNC Chapel Hill - School of Medicine, Surgery dept, Chapel Hill, NC; UNC Chapel Hill Jaycee Burn Center, Chapel Hill, NC; NC Jaycee Burn Center, Chapel Hill, NC; UNC Chapel Hill. NC, Chapel Hill, NC; The University of North Carolina at Chapel Hill, Chapel Hill, NC; University of North Carolina, Durham, NC; University of North Carolina, Chapel Hill, NC; University of North Carolina Chapel Hill, carrboro, NC; UNC Chapel Hill - School of Medicine, Surgery dept, Chapel Hill, NC; UNC Chapel Hill Jaycee Burn Center, Chapel Hill, NC; NC Jaycee Burn Center, Chapel Hill, NC; UNC Chapel Hill. NC, Chapel Hill, NC; The University of North Carolina at Chapel Hill, Chapel Hill, NC; University of North Carolina, Durham, NC; University of North Carolina, Chapel Hill, NC; University of North Carolina Chapel Hill, carrboro, NC; UNC Chapel Hill - School of Medicine, Surgery dept, Chapel Hill, NC; UNC Chapel Hill Jaycee Burn Center, Chapel Hill, NC; NC Jaycee Burn Center, Chapel Hill, NC; UNC Chapel Hill. NC, Chapel Hill, NC; The University of North Carolina at Chapel Hill, Chapel Hill, NC; University of North Carolina, Durham, NC; University of North Carolina, Chapel Hill, NC; University of North Carolina Chapel Hill, carrboro, NC; UNC Chapel Hill - School of Medicine, Surgery dept, Chapel Hill, NC; UNC Chapel Hill Jaycee Burn Center, Chapel Hill, NC; NC Jaycee Burn Center, Chapel Hill, NC; UNC Chapel Hill. NC, Chapel Hill, NC; The University of North Carolina at Chapel Hill, Chapel Hill, NC; University of North Carolina, Durham, NC; University of North Carolina, Chapel Hill, NC; University of North Carolina Chapel Hill, carrboro, NC; UNC Chapel Hill - School of Medicine, Surgery dept, Chapel Hill, NC; UNC Chapel Hill Jaycee Burn Center, Chapel Hill, NC; NC Jaycee Burn Center, Chapel Hill, NC; UNC Chapel Hill. NC, Chapel Hill, NC; The University of North Carolina at Chapel Hill, Chapel Hill, NC; University of North Carolina, Durham, NC; University of North Carolina, Chapel Hill, NC; University of North Carolina Chapel Hill, carrboro, NC; UNC Chapel Hill - School of Medicine, Surgery dept, Chapel Hill, NC; UNC Chapel Hill Jaycee Burn Center, Chapel Hill, NC; NC Jaycee Burn Center, Chapel Hill, NC; UNC Chapel Hill. NC, Chapel Hill, NC; The University of North Carolina at Chapel Hill, Chapel Hill, NC; University of North Carolina, Durham, NC; University of North Carolina, Chapel Hill, NC; University of North Carolina Chapel Hill, carrboro, NC; UNC Chapel Hill - School of Medicine, Surgery dept, Chapel Hill, NC; UNC Chapel Hill Jaycee Burn Center, Chapel Hill, NC; NC Jaycee Burn Center, Chapel Hill, NC; UNC Chapel Hill. NC, Chapel Hill, NC; The University of North Carolina at Chapel Hill, Chapel Hill, NC; University of North Carolina, Durham, NC; University of North Carolina, Chapel Hill, NC; University of North Carolina Chapel Hill, carrboro, NC; UNC Chapel Hill - School of Medicine, Surgery dept, Chapel Hill, NC; UNC Chapel Hill Jaycee Burn Center, Chapel Hill, NC; NC Jaycee Burn Center, Chapel Hill, NC; UNC Chapel Hill. NC, Chapel Hill, NC; The University of North Carolina at Chapel Hill, Chapel Hill, NC; University of North Carolina, Durham, NC; University of North Carolina, Chapel Hill, NC; University of North Carolina Chapel Hill, carrboro, NC; UNC Chapel Hill - School of Medicine, Surgery dept, Chapel Hill, NC; UNC Chapel Hill Jaycee Burn Center, Chapel Hill, NC; NC Jaycee Burn Center, Chapel Hill, NC; UNC Chapel Hill. NC, Chapel Hill, NC; The University of North Carolina at Chapel Hill, Chapel Hill, NC

## Abstract

**Introduction:**

There is an increased incidence of renal failure in the expanding aging population. However, the effect of renal failure as a co-morbid condition on burn mortality remains overlooked in conventional predictive models. Our retrospective analysis of burn survivors and non-survivors aims to critically evaluate management of burn patients with pre-existing renal failure.

**Methods:**

We conducted a retrospective analysis utilizing data from the Institutional Burn Center registry, which was linked to clinical and administrative records. Our study included all adult patients admitted to the Burn Center between January 1, 2012, and December 31, 2022. We collected and analyzed various parameters, including patient demographics, length of hospital stay (LOS), comorbid conditions, and mortality outcomes. We evaluated the association between renal failure and burn mortality using logistic regression modeling.

**Results:**

Over the ten-year study period, a total of 9,955 adult patients were admitted to the BICU. 124 patients presented with a diagnosis of renal failure: 18 non-survivors, 106 survivors. Our analysis revealed that renal failure was associated with a nearly three-fold increase in the odds of mortality (OR 2.64, CI 1.33-5.01, p=0.004) when accounting for factors present in traditional predictive models such as burn size, age, and inhalational injury. Additionally, renal failure was identified as a co-morbidity in 6% of all deaths for this time frame and 14.5% of patients with this diagnosis on admission are deceased. These findings underscore the significant impact of renal failure on burn patient outcomes.

**Conclusions:**

As our population continues to age, burn providers will increasingly encounter the complex challenge of managing an acute burn with multiple comorbidities. Our study highlights the intuitive understanding that patients with pre-existing renal failure have a heightened mortality risk when suffering from an acute burn injury. In light of this, it is imperative to consider potential adjustments to burn resuscitation and care protocols for this at-risk population, with the ultimate goal of enhancing the chances of survival to hospital discharge.

**Applicability of Research to Practice:**

This study emphasizes the pressing need for further research into the effects of multiple comorbidities on mortality among burn patients. The insights gained from this research have practical implications for refining clinical approaches and optimizing care strategies for this vulnerable patient population.

